# Meaning Guides Attention during Real-World Scene Description

**DOI:** 10.1038/s41598-018-31894-5

**Published:** 2018-09-10

**Authors:** John M. Henderson, Taylor R. Hayes, Gwendolyn Rehrig, Fernanda Ferreira

**Affiliations:** 1 Center for Mind and Brain, University of California, Davis, USA; 2 Department of Psychology, University of California, Davis, USA

## Abstract

Intelligent analysis of a visual scene requires that important regions be prioritized and attentionally selected for preferential processing. What is the basis for this selection? Here we compared the influence of meaning and image salience on attentional guidance in real-world scenes during two free-viewing scene description tasks. Meaning was represented by meaning maps capturing the spatial distribution of semantic features. Image salience was represented by saliency maps capturing the spatial distribution of image features. Both types of maps were coded in a format that could be directly compared to maps of the spatial distribution of attention derived from viewers’ eye fixations in the scene description tasks. The results showed that both meaning and salience predicted the spatial distribution of attention in these tasks, but that when the correlation between meaning and salience was statistically controlled, only meaning accounted for unique variance in attention. The results support theories in which cognitive relevance plays the dominant functional role in controlling human attentional guidance in scenes. The results also have practical implications for current artificial intelligence approaches to labeling real-world images.

## Introduction

The visual world presents us with far more information than we can process at any given moment. Intelligent analysis of a scene therefore requires selection of relevant scene regions for prioritized processing. How does the brain select those aspects of the world that should receive priority for visual and cognitive analysis? A key mechanism of selection in natural vision is overt visual attention via saccadic eye movements^1–8^. What we see, comprehend, and remember about the visual world is a direct result of this prioritization and selection. A central topic in visual cognition, therefore, is understanding how overt attention is guided through scenes.

Visual saliency theory has provided an influential approach to attentional guidance in real world scenes. The theory proposes that attention is strongly controlled by contrasts in low-level image features such as luminance, color, and edge orientation^9,10^. In visual saliency theory, saliency maps are generated by pooling contrasts in these semantically uninterpreted features, and attention is then assumed to be captured or “pulled” to visually salient scene regions^11–16^. In this view, the scene image in a sense prioritizes its own regions for attentional selection. The appeal of image guidance theory is that visual salience is both neurobiologically inspired in that the visual system is known to compute these features, and computationally tractable in that working models have been implemented that can generate saliency from these known neurobiological processes^4^.

In contrast to saliency theory, cognitive guidance theory emphasizes the strong role of meaning in directing attention in scenes. In this view, attention is “pushed” by the cognitive system to scene regions that are semantically informative and cognitively relevant in the current situation^17^. Cognitive guidance theory places explanatory primacy on scene semantics interacting with viewer goals^6,18–25^. For example, in the cognitive relevance model^26,27^, the priority of a scene entity or region for attention is determined by its meaning in the context of the scene and the current goals of the viewer. In this view, meaning determines attentional priority, with image properties used only to generate the set of potential attentional targets but not their priority for selection. Attentional priority is then assigned to those potential targets based on knowledge representations generated from scene analysis and memory. The visual stimulus remains important in that it is used to parse the scene into a map of potential targets, and processes related to image differences (i.e., those captured by saliency models) likely play a role in which targets are encoded in the map. But the critical hypothesis is that this parse generates a flat (that is, unranked) landscape of potential attentional targets and not a landscape ranked by salience. Knowledge representations then provide the attentional priority ranking for attentional targets based on their meaning^3,26,27^.

To investigate and directly compare the influences of image salience and meaning on attention, it is necessary to represent both in a manner that can generate comparable quantitative predictions. Contrasting predictions can then be tested against attention. Implemented computational saliency models output a spatial map of saliency values over a scene, providing such quantitative predictions^11,12,28–30^. How can we similarly generate a spatial distribution of meaning over a scene for comparison to saliency? Unfortunately, it is far more difficult to create a computational model of meaning than it is to create a computational model of image salience, a likely reason that saliency models have been so popular^4^.

**Figure 1:**
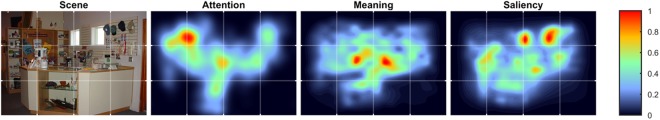
An example scene with its attention map empirically determined from viewer fixations, along with its meaning and saliency maps. (Grid lines are included for ease of comparison and were not present in the scenes).

To address this issue, we have recently developed a *meaning map* representation of the spatial distribution of meaning across scenes^31^. The key idea of a meaning map is that it captures the spatial distribution of semantic content in the same format that a saliency map captures the spatial distribution of image salience. To create meaning maps, we used crowd-sourced responses given by naïve subjects who rated the meaningfulness of a large number of context-free scene patches. Photographs of real-world scenes were divided into dense arrays of objectively defined circular overlapping patches at two spatial scales. These patches were then presented to raters on Mechanical Turk independently of the scenes from which they were taken. The raters indicated how meaningful they judged each patch to be. We constructed smoothed maps for each scene by interpolating these ratings over a large number of patches and raters for each scene. Like image salience, meaning is spatially distributed non-uniformly across scenes, with some scene regions relatively rich in semantic content and others relatively sparse (see Fig. 1).

A meaning map provides the conceptual analog of a saliency map by representing the spatial distribution of semantic features (rather than image features) across a scene. Meaning maps therefore provide a basis for directly comparing the influences of meaning and salience on attentional guidance, using the same methods that have been used to test the goodness of fit of predictions from saliency theory^31^.

In our first study comparing the roles of meaning and image salience, subjects were engaged in a viewing task in which they freely viewed scenes to prepare for a task (memory or aesthetic judgement) that was presented after the scene was removed from view^31^. It could be that under such conditions, viewers do not guide attention as quickly or in as controlled a manner as they might in an on-line task requiring responses in real time during viewing. Here we set out to determine how well meaning and image salience predict attentional guidance in free viewing of scenes when the viewer is actively engaged with and responding to each scene on-line, and attention is directly relevant to the on-line task.

We report the results of two experiments using two different free-viewing scene description tasks. Most theories of language production assume that production is at least moderately incremental^32,33^, so that speakers dynamically interleave planning and speaking rather than preparing the entire description in advance of any articulation. Because production is incremental, small phrasal-sized units of speech are planned in response to fixations made to different scene regions. Scene description therefore allows us to assess the influence of image salience versus meaning in a continuous, on-line task in which attention to specific scene regions is functional and necessary. In Experiment 1, subjects were asked to describe what someone might do in each scene. In Experiment 2, subjects were simply asked to describe each scene. In both experiments, subjects were instructed to begin their description as soon as each scene appeared and to continue speaking for 30 seconds. Eye movements over the scenes were tracked throughout the trial.

In summary, the goal of this study was to test current theoretical approaches to attentional guidance in real-world scenes across two free-viewing experiments using two scene description tasks. We applied a recently developed method, meaning maps, to generate the spatial distribution of semantic content across the scenes, and used a popular computational model to generate image salience. We then tested cognitive and image guidance theories by comparing the relative success of meaning maps and saliency maps to predict the distribution of attention as assessed by eye fixations in the scene description tasks.

## Experiment 1

In Experiment 1, subjects were asked to describe a set of actions that could be performed in the currently viewed scene. Each scene was presented for 30 s, and both eye movements and speech were recorded throughout viewing.

## Results

On average, subjects began speaking 2.500 s (SE = 46 ms) after scene onset, and stopped speaking 26.178 s (SE = 181 ms) after scene onset. The delay prior to speaking reflects speech preparation and planning referred to as an initial apprehension stage^33^, whereas the tendency to stop speaking shortly before the end of the trial reflected the subject’s decision that they had fully described all actions in the scene. Because the focus of the experiment was on attentional guidance during an on-line viewing task, only eyetracking data up to the end of speech in each trial was included in the attention map analyses.

Meaning maps and saliency maps served as predictions concerning how viewers distributed their attention over scenes. Attention maps representing the distribution of attention were generated from eye fixations. Figure 1 shows examples of these maps. The critical empirical question is how well the two types of prediction maps align with the attention maps in this on-line scene description task. To answer this question, we used linear correlation to quantify the degree to which the meaning maps and saliency maps accounted for shared and unique variance in the attention maps for each scene^31^. Linear correlation is a map-level analysis method that makes relatively few assumptions, is intuitive, can be visualized, and generally balances the various positives and negatives of different analysis approaches^34^. It also provides a sensitive method for teasing apart variance in the attention maps accounted for by meaning and image salience.

**Figure 2:**
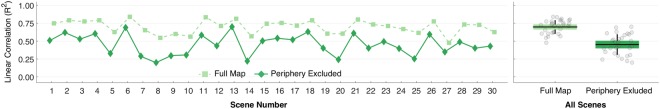
Correlation (R^2^) between saliency and meaning maps. The line plots show the correlation between the meaning and saliency maps for each scene. The scatter box plots on the right show the corresponding grand correlation mean across N = 30 scenes (black horizontal line), 95% confidence intervals (colored box), and 1 standard deviation (black vertical line). The top (light) line shows the full correlation and the bottom line shows the correlation with center bias removed. The mean correlations differed significantly from zero, p < 0.0001.

We started by examining the correlation between the meaning and saliency maps themselves (Fig. 2). First, we computed the overall correlation across the two maps for each scene. The squared correlation (R^2^) between the full meaning saliency maps was 0.70, averaged across the 30 scenes (SD = 0.10), t(29) = 39.92, p < 0.0001, 95% CI [0.66 0.73]. However, this correlation overestimated the strength of the relationship because of the shared center bias present in both maps: These maps all had values of zero in the periphery, artificially increasing the correlations. Therefore, we also computed the correlations between the meaning and saliency maps excluding the periphery of each map to provide a more accurate estimate of the true relationship. The R^2^ excluding the periphery was 0.45 (SD = 0.14), t(29) = 17.16, p < 0.0001, 95% CI [0.40 0.51]. The strength of the correlation across maps is consistent with previous work^31^ and with the proposal that the success of image salience in accounting for attention in previous studies could in fact be due to the correlation of image salience with meaning^26,27^. At the same time, these correlations leave considerable unique variance in meaning and image salience that could be independently associated with the distribution of attention.

**Figure 3:**
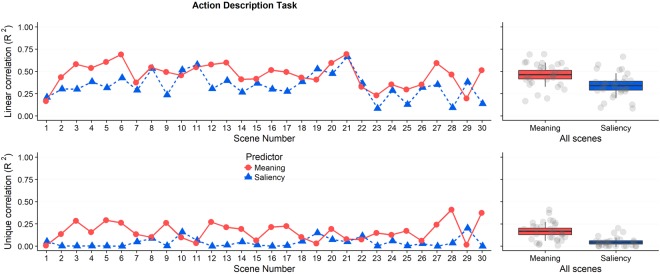
Squared linear correlation and semi-partial correlation by scene and across all scenes in Experiment 1. The line plots show the linear correlation (top) and semi-partial correlation (bottom) between fixation density and meaning (red) and salience (blue) by scene. The scatter box plots on the right show the corresponding grand mean (black horizontal line), 95% confidence intervals (colored box), and 1 standard deviation (black vertical line) for meaning and salience across all 30 scenes.

Our primary concern in the present study involved the relationships between the theory-based maps (meaning and saliency) and the observed attention maps. Fig. 3 presents these data for each of the 30 scenes. Each data point shows the relationship (R^2^) between the meaning map and the observed attention map by scene (red), and between the saliency map and the observed attention map by scene (blue). The top half of Fig. 3 shows the squared linear correlations. On average across the 30 scenes, meaning accounted for 46% of the variance in fixation density (M = 0.46, SD = 0.14) and salience accounted for 34% of the variance in fixation density (M = 0.34, SD = 0.14). A two-tailed t-test revealed the advantage for meaning over salience to be statistically significant, t(58) = 3.47, p = 0.001, 95% CI [0.052 0.194].

The critical theoretical question is how well the unshared variance in meaning and salience predicted the distribution of attention. To examine the unique variance in attention explained by meaning and salience when controlling for their shared variance, we computed squared semi-partial correlations (bottom half of Fig. 3). Across the 30 scenes, meaning accounted for a significant 17% additional variance in attention after controlling for salience (M = 0.17, SD = 0.10), whereas salience accounted for a non-significant 4% additional variance after controlling for meaning (M = 0.04, SD = 0.05). A two-tailed t-test confirmed that this difference was statistically significant, t(58) = 5.766, p < 0.0001, 95% CI [0.081 0.166]. These results indicate that meaning explained the distribution of attention over scenes better than image salience.

It has sometimes been proposed that attention might initially be guided by image salience, but that as the scene is viewed over time, meaning begins to play a greater role in attentional guidance^7,35–38^. To test this hypothesis, we conducted time-step analyses. Linear correlation and semi-partial correlations were generated based on a series of attention maps, with each map representing sequential eye fixations (i.e., 1st, 2nd, 3rd fixation) in each scene. We then compared these time-step attention maps to the meaning and saliency maps to test whether the relative importance of meaning and salience in predicting attention changed over time.

Linear and semi-partial correlations were generated based on a series of attention maps, with each map representing sequential eye fixations. We then compared the first three time-step attention maps to the meaning and saliency maps to test whether the relative importance of meaning and salience in predicting attention changed over time. For the linear correlations, the relationship was stronger between the meaning and attention maps for all time-steps, and was highly consistent across the 30 scenes. Contrary to the saliency first hypothesis, meaning accounted for 35.2%, 20.4%, and 16.3% of the variance in the first three fixations, respectively, whereas salience accounted for 11.0%, 12.5%, and 10.3% of the variance in these fixations. Two sample two-tailed t-tests were performed for these three fixations, and p-values were corrected for multiple comparisons using the false discovery rate (FDR) correction^39^. This procedure confirmed the advantage for meaning over salience during the first three fixations (FDR <0.05).

Importantly, the improvement in R^2^ for the meaning maps over saliency maps observed in the linear correlation analyses was also found across fixations in the partial correlations (FDR < 0.05), with meaning accounting for 27.1%, 13.6%, and 9.9% of the unique variance in the first three fixations, whereas salience accounted for just 2.8%, 5.7%, and 3.9% of the unique variance in the first three fixations, respectively. Overall, counter to the salience-first hypothesis and consistent with our prior work^31^, meaning accounted for more variance in attention than salience beginning with the very first fixation. Indeed, contrary to salience-first, the meaning advantage was quantitatively larger for the first fixation than for subsequent fixations.

## Experiment 2

It could be that in Experiment 1, the focus on describing potential activities biased viewers to attend to functions afforded by scenes rather their visual properties, which may have biased attention toward meaningful rather than visually salient features, leading to a stronger relationship between meaning and attention. If this is correct, then a more open-ended description task should lead to greater reliance on image salience. To test this hypothesis, In Experiment 2 we replicated and extended the basic scene description paradigm by asking subjects to describe the scenes in whatever manner they liked. These more open-ended instructions should be less constraining and so should eliminate any implicit bias to attend to meaning related to scene function. As in Experiment 1, each scene was presented for 30 s during the description task and both eye movements and speech were recorded throughout each trial.

## Results

**Figure 4:**
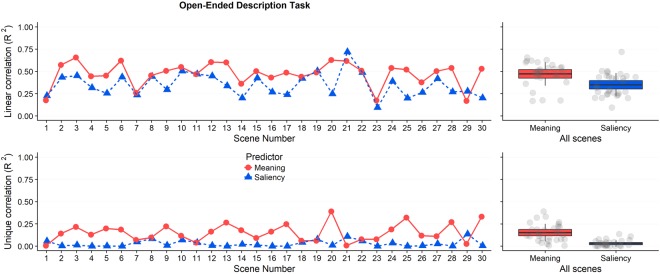
Squared linear correlation and semi-partial correlation by scene and across all scenes in Experiment 2. The line plots show the linear correlation (top) and semi-partial correlation (bottom) between fixation density and meaning (red) and salience (blue) by scene. The scatter box plots on the right show the corresponding grand mean (black horizontal line), 95% confidence intervals (colored box), and 1 standard deviation (black vertical line) for meaning and salience across all 30 scenes.

Figure 4 presents the primary data for each of the 30 scenes. Each data point shows the relationship (R^2^) between the meaning map and the observed attention map by scene (red), and between the saliency map and the observed attention map by scene (blue). The top half of Fig. 4 shows the squared linear correlations. On average across the 30 scenes, meaning accounted for 47% of the variance in fixation density (M = 0.47, SD = 0.13) and salience accounted for 35% of the variance in fixation density (M = 0.35, SD = 0.13). A two-tailed t-test revealed that the advantage for meaning over salience was statistically significant, t(58) = 3.61, p < 0.001, 95% CI [0.055 0.191]. These results replicate those of Experiment 1.

Once again, the critical question was how well unshared variance in meaning and salience predicted the distribution of attention. The squared semi-partial correlations for meaning and salience controlling for their shared variance are presented in the bottom half of Fig. 4. Across the 30 scenes, meaning accounted for a significant 15% additional variance in the attention maps after controlling for salience (M = 0.15, SD = 0.10), whereas salience accounted for a non-significant 3% additional variance after controlling for meaning (M = 0.03, SD = 0.04). A two-tailed t-test confirmed that this difference was statistically significant, t(58) = 6.390, p < 0.0001, 95% CI [0.084 0.162]. These results also replicate Experiment 1 and indicate that meaning explained the distribution of attention over scenes better than salience.

To investigate whether the importance of salience and meaning changed over the course of viewing, we again conducted a time-step analysis as described in Experiment 1. For the linear correlations, the relationship was stronger between the meaning and attention maps for all time-steps and was highly consistent across the 30 scenes. Contrary to the saliency first hypothesis, meaning accounted for 38.2%, 22.3%, and 17.0% of the variance in the first 3 fixations, whereas salience accounted for only 11.5%, 10.8%, and 13.0% of the variance in the first 3 fixations, respectively. Two sample two-tailed t-tests corrected for multiple comparisons using the false discovery rate (FDR) correction confirmed the advantage for meaning over salience during the first 2 time-steps (FDR <0.05).

The improvement in R^2^ for the meaning maps over saliency maps observed in the overall analyses was also found in the partial correlations for the first 2 time-steps (FDR < 0.05), and the advantage was marginal for the third time-step (FDR* = *0.08), with meaning accounting for 29.9%, 15.3%, and 9.0% of the unique variance in the first 3 fixations, whereas salience accounted for 3.2%, 3.8%, and 5.1% of the unique variance in the first 3 fixations, respectively. Again, counter to the salience-first hypothesis, meaning accounted for more variance in attention than salience beginning with the very first fixation.

## Discussion

Real-world scenes contain an enormous amount of information, but human vision and visual cognition are capacity-limited: Only a small amount of the available information can be processed at any given moment. Efficient visual analysis therefore requires properly selecting the information that is most relevant given the current needs of the viewer.

How is attention guided through real-world scenes? The concept of image salience, typically represented by computationally derived saliency maps, has provided an important theoretical framework for thinking about the guidance of attention in scenes^11–16^. At the same time, it is well established that people are highly sensitive to the semantic content of the visual world they perceive^5,6^, suggesting that attention is directed by the semantic content of a scene rather than by visually salient image features^26,27^. Until recently it has been difficult to directly contrast image salience and meaning so that their relative influences on attention can be compared. To address this challenge, we developed a method for identifying and representing the spatial distribution of meaning in real world scene images^31^. The resulting meaning maps quantify the spatial distribution of semantic content across scenes in the same format that saliency maps quantify the spatial distribution of image salience. Meaning maps therefore provide a method for directly comparing influences of the distributions of meaning and image salience. In the present study, we used meaning maps to test the relative importance of meaning and salience by assessing theoretically motivated meaning maps and saliency maps against observed attention maps generated from the eye movements of viewers during two scene description experiments.

In Experiment 1, subjects described actions that could take place in the scenes. In Experiment 2, they simply described the scenes. In both experiments, the main results were that meaning accounted for more of the overall variance and more of the unique variance in attention compared to image salience. The spatial distribution of meaning and image salience were correlated across the images, but importantly, when this correlation was statistically controlled, meaning accounted for substantial additional variance in attention, whereas salience did not. Furthermore, the advantage for meaning started with the very first subject-determined fixation. Together these results support cognitive over image salience theories of attentional guidance.

As an example of how cognitive guidance might work, the cognitive relevance model^26,27^ proposes that a representation of scene entities is generated based on an initial image parse, with attentional priority assigned to entities (e.g., perceptual objects) based on their meaning and relevance in the context of the current task. In this view, contrasts in image features are not used to assign attentional priority, but instead serve as the basis of the scene parse. It is important to note that cognitive guidance does not require that meaning be assigned simultaneously to all perceptually mapped objects across the entire scene. That is, cognitive guidance does not require a strong late-selection view of scene perception. Indeed, there is significant evidence against semantic analysis of objects in the visual periphery of scenes^40–43^. Instead, when a scene is initially encountered, its gist can be quickly apprehended^44–47^. Apprehending the gist allows access to schema representations that provide constraints on what objects are likely to be present and where those objects are likely to be located within the scene^3,7^, and can guide attention at the very earliest points of scene viewing^7,48–50^. Information retrieved from memory schemas can be combined with low-quality visual information from the periphery to assign tentative meaning to perceptual objects and other scene regions. These initial representations provide a rich set of priors and can be used to generate predictions for guiding attention to regions that have not yet been identified^4,30^. In addition, many saccades during scene viewing are relatively short in amplitude, such that attention is frequently guided from one location to the next using parafoveal and perifoveal information relatively close to the fovea where at least partial identity and meaning can be determined.

The type of scene meaning we have investigated here is based on ratings of scene patches that were shown to raters independently of their scenes and independently of any task or goal other than the rating itself. These experiments therefore investigated the role of what we call *scene-intrinsic context-free meaning* on attention. We can contrast this with other types of meaning based on other types of cognitive representations. As one example, we might consider *scene-intrinsic contextualized meaning*, in which the degree of meaning associated with a scene region is based on its relationship to the meaning of the scene as a whole. For instance, an octopus might be meaningful floating in an undersea scene, but it might be even more meaningful in a farmyard scene or floating in the sky^7,40,42,51–54^. Note that in all cases, a scene parse will lead to a representation of an octopus object, but its meaning value may differ substantially. Similarly, we might consider *task-related* and *goal-related contextualized meaning*, in which the meaning of a scene region is based on its relationship to the viewer’s task and goals rather than intrinsic to the scene itself. For example, during a tennis serve, the bounce point of the ball might not be especially intrinsically meaningful, but might become meaningful right after the ball leaves the server’s racket given the goal of determining where the ball is going to end up^1,2,4,25,55,56^. How cognitive representations that encode these types of meaning interact with each other to guide attention is currently unknown. The meaning map approach provides a basis for pursuing these questions, and the present results suggest that such a pursuit would be worthwhile. Note that given these considerations, the present study using context-free meaning potentially employed the least potent version of meaning maps, and we may therefore be underestimating the degree to which meaning predicts attention.

Given the dominance of meaning over image salience we have observed previously^31^ and in the present study, how do we account for prior results suggesting that image salience can account for attention? One answer is that in some earlier studies, meaningless images (e.g., fractals) were used as stimuli. In these cases, the only meaning to be found in the image may be feature contrasts. That is, salience may be meaningful when no other type of meaning is available. In the case of studies supporting an effect of image salience in meaningful real-world scenes, it is likely that salience accounts for attention to some degree because of its strong correlation with meaning^31,57^. As we have shown here, shared variance between meaning and salience was about .70 overall and .45 when the down-weighted map peripheries were removed from the analysis. Given this relationship, the only way to unambiguously demonstrate an influence of salience over meaning is to de-correlate them. And as we have shown, when this is done statistically, there is little evidence for an influence of image salience independent of meaning both in the present task and in scene memorization and aesthetic judgement tasks^31,58^.

## Conclusion

In this study we investigated the relative importance of meaning and image salience on the guidance of attention when viewers engage in on-line free-viewing tasks involving real-world scene description. To assess meaning, we used meaning maps, a recently developed method for measuring and representing the distribution of semantic content over scene images^31^. We assessed the relative influences of meaning and image salience in two language production tasks, one in which subjects simply described the scenes, and a second in which they described scene functions. We found that the spatial distribution of meaning was better able than image salience to account for the guidance of attention in both tasks. The pattern of results is consistent with cognitive control theories of scene viewing in which attentional priority is assigned to scene regions based on semantic information value rather than image salience. In addition to the important theoretical implications of these findings for understanding attention, the present results also have important implications for current artificial intelligence and machine learning approaches to detecting and labeling real-world images. These systems often use image salience to locate regions likely to be attended by people, and the present results suggest that such an approach may be limited.

## Methods

### Meaning and Image Saliency Map Generation

We created meaning maps based on the method developed in our previous work^31^. Each 1024 × 768 pixel scene image was decomposed into a series of partially overlapping (tiled) circular patches at two spatial scales. The full patch stimulus set consisted of 12000 unique fine-scale patches (87 pixel diameter) and 4320 unique coarse-scale patches (205 pixel diameter) for a total of 16320 scene patches. Scene patch ratings were performed by 165 subjects on Amazon Mechanical Turk. Subjects were recruited from the United States, had a hit approval rate of 99% and 500 hits approved, and were only allowed to participate in the study once. Subjects were paid $0.50 cents per assignment and all subjects provided informed consent.

Each subject rated 300 random patches extracted from the scenes. Subjects were instructed to assess the meaningfulness of each patch based on how informative or recognizable they thought it was. Subjects were first given examples of two low-meaning and two high-meaning scene patches to make sure they understood the rating task, and then rated the meaningfulness of scene patches on a 6-point Likert scale (‘very low’,‘low’,‘somewhat low’,‘somewhat high’,‘high’,‘very high’). Patches were presented in random order and without scene context, so ratings were based on context-free judgments. Each unique patch was rated three times by three independent raters for a total of 48960 ratings. Due to the high degree of overlap across patches, each fine-scale patch contained rating information from 27 independent raters and each coarse-scale patch contained rating information from 63 independent raters. Meaning maps were generated from the ratings by averaging, smoothing, and then combining scale maps from the corresponding patch ratings. The ratings for each pixel at each scale in each scene were averaged, producing an average fine-scale and coarse-scale rating map for each scene. The average rating maps were then smoothed using thin-plate spline interpolation (Matlab‘fit’ using the‘thinplateinterp’ method). Finally, the smoothed maps were combined using a simple average. This procedure resulted in a meaning map for each scene. The final maps were blurred using a Gaussian kernel followed by a multiplicative center bias operation that down-weighted the scores in the periphery to account for the central fixation bias commonly observed in scene viewing in which subjects concentrate their fixations more centrally and rarely fixate the outside border of a scene^14,27,59^.

Image-based saliency maps were computed for each scene using the Graph-based Visual Saliency (GBVS) toolbox with default settings^15^. GBVS is a prominent saliency model that combines maps of neurobiologically inspired low-level image features. A center bias operation is intrinsic to the GBVS saliency maps.

Meaning and saliency maps were normalized to a common scale using image histogram matching, with the fixation map for each scene serving as the reference image for the corresponding meaning and saliency maps. Histogram matching of the meaning and saliency maps was accomplished using the Matlab function ‘imhistmatch’ in the Image Processing Toolbox. Further details about map generation can be found in previous work^31^.

## Experiment 1

### Subjects

University of California, Davis, undergraduate students with normal or corrected-to-normal vision participated in the experiment. All subjects were naive concerning the purposes of the experiment and provided informed consent. An initial group of 32 subjects was recruited, and data from an *a priori* target number of 30 subjects were included in the analyses. The target of 30 was chosen by examining in several independent data sets the number of subjects needed to establish stable attention maps. The two excluded subjects could not be accurately eye-tracked.

### Stimuli

Thirty digitized (1024 × 768 pixels) photographs of real world scenes depicting a variety of indoor and outdoor environments were presented. Of the 30 scenes, 10 depicted outdoor environments and 20 depicted indoor environments. The outdoor scenes included various buildings (e.g., houses, businesses), and half (5) of the outdoor scenes were street views. The set of indoor scenes included 5 living rooms, 3 kitchens, 2 desk areas within a room, and 10 different room types. The scenes did not contain people. Twenty of these stimuli were included in Henderson and Hayes (2017) and ten had not been tested previously. Scenes were selected to maximize the absolute difference between the meaning and saliency maps to enable separation of the influence of each on visual attention. In addition, the selected scenes were easy to describe in that they contained recognizable and readily distinguishable objects in familiar contexts.

### Apparatus

Eye movements were recorded with an SR Research EyeLink 1000+ tower mount eyetracker (spatial resolution 0.01) sampling at 1000 Hz. Subjects sat 90 cm away from a 21” monitor, so that scenes subtended approximately 33**°** × 25**°** of visual angle at 1024 × 768 pixels. Head movements were minimized using a chin and forehead rest. Although viewing was binocular, eye movements were recorded from the right eye. The experiment was controlled with SR Research Experiment Builder software. Audio was digitally recorded using a Roland Rubix 22 USB audio interface and a Shure SM86 cardioid condenser microphone.

### Procedure

Each trial began with a five-point array of fixation crosses used to check calibration. The subject then fixated the center cross until the experimenter pressed a key to begin the trial. The scene was then presented for 30 s while eye movements and speech were recorded. A unique pseudo-random scene order was generated for each subject with the restriction that two scenes of the same category (e.g., kitchen) did not occur consecutively. Subjects were instructed to describe a set of actions that could be performed in the scene in the following way: “In this experiment, you will see a series of scenes. In each scene, think of the average person. Describe what the average person would be inclined to do in the scene. You will have 30 seconds to respond.” After the 30 second response window terminated, subjects proceeded to the next trial by pressing any button on a response box. Prior to the experimental trials, subjects completed three practice trials to familiarize themselves with the task and the duration of the response period.

A calibration procedure was performed at the start of each session to map eye position to screen coordinates. Successful calibration required an average error of less than 0.49° and a maximum error of less than 0.99°. Fixations and saccades were segmented with EyeLink’s standard algorithm using velocity and acceleration thresholds (30°/s and 9500°/s^2^).

Eye movement data were imported offline into Matlab using the EDFConverter tool. The first fixation, determined by the fixation cross and always located at the center of the display, was eliminated from analysis.

## Experiment 2

### Subjects

Thirty University of California, Davis, undergraduate students with the characteristics listed in Experiment 1 participated in this experiment. None of the subjects participated in Experiment 1. An initial group of 38 subjects was recruited, and data from an *a priori* target number of 30 subjects who met the inclusion criteria were included in the analyses. Excluded subjects failed to calibrate (one), did not follow instructions to speak out loud (one), or could not be accurately eye-tracked (six).

### Stimuli, Apparatus

Stimuli and apparatus were the same as in Experiment 1.

### Procedure

The procedure was the same as in Experiment 1, with the exception that the instructions were changed such that subjects were instructed simply to describe each scene: “In this experiment, you will see a series of scenes. You will have 30 seconds to describe the scene out loud.”

## Data Availability

The datasets generated during and/or analysed during the current study are available from the corresponding author on reasonable request.

## References

[1] Land MF, Hayhoe MM (2001). In what ways do eye movements contribute to everyday activities?. Vision Research.

[2] Hayhoe MM, Ballard D (2005). Eye movements in natural behavior. Trends in Cognitive Sciences.

[3] Henderson JM (2003). Human gaze control during real-world scene perception. Trends in Cognitive Sciences.

[4] Henderson JM (2017). Gaze Control as Prediction. Trends in Cognitive Sciences.

[5] Buswell, G. T. *How People Look at Pictures*. (University of Chicago Press Chicago, 1935).

[6] Yarbus, A. L. *Eye movements and vision*. 10.1016/0028-3932(68)90012-2 (Plenum Press, 1967).

[7] Henderson JM, Hollingworth A (1999). High-level scene perception. Annual Review of Psychology.

[8] Rayner K (2009). The 35th Sir Frederick Bartlett Lecture: Eye movements and attention in reading, scene perception, and visual search. The Quarterly Journal of Experimental Psychology.

[9] Treisman AM, Gelade G (1980). A Feature-Integration Theory of Attention. Cognitive Psychology.

[10] Wolfe JM, Horowitz TS (2017). Five factors that guide attention in visual search. Nature Human Behaviour.

[11] Itti L, Koch C (2001). Computational modelling of visual attention. Nature Reviews Neuroscience.

[12] Parkhurst D, Law K, Niebur E (2002). Modelling the role of salience in the allocation of visual selective attention. Vision Research.

[13] Borji A, Parks D, Itti L (2014). Complementary effects of gaze direction and early saliency in guiding fixations during free viewing. Journal of Vision.

[14] Borji A, Sihite DN, Itti L (2013). Quantitative analysis of human-model agreement in visual saliency modeling: A comparative study. IEEE Transactions on Image Processing.

[15] Harel, J., Koch, C. & Perona, P. Graph-Based Visual Saliency. *Advances in neural information processing systems*. 1–8, doi:10.1.1.70.2254 (2006).

[16] Koch C, Ullman S (1985). Shifts in Selective Visual Attention: Towards the Underlying Neural Circuitry. Human Neurobiology.

[17] Henderson JM (2007). Regarding scenes. Current Directions in Psychological Science.

[18] Einhäuser W, Rutishauser U, Koch C (2008). Task-demands can immediately reverse the effects of sensory-driven saliency in complex visual stimuli. Journal of vision.

[19] Tatler BW, Hayhoe MM, Land MF, Ballard DH (2011). Eye guidance in natural vision: Reinterpreting salience. Journal of Vision.

[20] Buswell, G. T. *How People Look at Pictures*. *Psychological Bulletin* (University of Chicago Press Chicago,). 10.1037/h0053409 (1936).

[21] Henderson, J. M. Eye movements and scene perception. In *The Oxford Handbook of* Eye *Movements* (eds Liversedge, S. P., Gilchrist, I. D. & Everling, S.) 593–606 (Oxford; New York: Oxford University Press, 2011).

[22] Wu CC, Wick FA, Pomplun M (2014). Guidance of visual attention by semantic information in real-world scenes. Frontiers in Psychology.

[23] Neider MB, Zelinsky G (2006). Scene context guides eye movements during visual search. Vision Research.

[24] Stirk JA, Underwood G (2007). Low-level visual saliency does not predict change detection in natural scenes. Journal of Vision.

[25] Hayhoe, M. M. V and Action. 389–413, 10.1146/annurev-vision-102016-061437 (2017).

[26] Henderson JM, Malcolm GL, Schandl C (2009). Searching in the dark: Cognitive relevance drives attention in real-world scenes. Psychonomic Bulletin & Review.

[27] Henderson, J. M., Brockmole, J. R., Castelhano, M. S. & Mack, M. Visual saliency does not account for eye movements during visual search in real-world scenes. In *Eye movements: A window on mind and brain* (eds Van Gompel, R. P. G., Fischer, M. H., Murray, Wayne, S. & Hill, R. L.) 537–562 (Elsevier Ltd), doi:10.1016/B978-008044980-7/50027-6 (2007).

[28] Itti L, Koch C, Niebur E (1998). A model of saliency-based visual attention for rapid scene analysis. Pattern Analysis and Machine Intelligence, IEEE Transactions on.

[29] Borji A, Itti L (2013). State-of-the-art in visual attention modeling. IEEE Transactions on Pattern Analysis and Machine Intelligence.

[30] Torralba A, Oliva A, Castelhano MS, Henderson JM (2006). Contextual guidance of eye movements and attention in real-world scenes: the role of global features in object search. Psychological Review.

[31] Henderson JM, Hayes TR (2017). Meaning-based guidance of attention in scenes as revealed by meaning maps. Nature Human Behaviour.

[32] Ferreira F, Swets B (2002). How incremental is language production? Evidence from the production of utterances requiring the computation of arithmetic sums. Journal of Memory and Language.

[33] Griffin ZM, Bock K (1999). What the eyes say about speaking. Psychological Science.

[34] Bylinskii, Z., Judd, T., Oliva, A., Torralba, A. & Durand, F. What do different evaluation metrics tell us about saliency models? *arXiv* 1–23 (2016). 10.1109/TPAMI.2018.2815601 29993800

[35] Mannan SK, Ruddock KH, Wooding DS (1996). The relationship between the locations of spatial features and those of fixations made during visual examination of briefly presented images. Spatial Vision.

[36] Parkhurst D, Law K, Niebur E (2002). Modeling the role of salience in the allocation of overt visual attention. Vision Research.

[37] Anderson NC, Ort E, Kruijne W, Meeter M, Donk M (2015). It depends on when you look at it: Salience influences eye movements in natural scene viewing and search early in time. Journal of Vision.

[38] Henderson, J. M. & Ferreira, F. Scene perception for psycholinguists. In *The Interface of language, Vision, and Action* (Psychology Press, 2004).

[39] Benjamini Y, Hochberg Y (1995). Controlling the false discovery rate: a practical and powerful approach to multiple testing. Journal of the Royal Statistical Society.

[40] Henderson JM, Weeks PAJ, Hollingworth A (1999). The effects of semantic consistency on eye movements during complex scene viewing. Journal of Experimental Psychology: Human Perception and Performance.

[41] Võ MLH, Henderson JM (2011). Object–scene inconsistencies do not capture gaze: evidence from the flash-preview moving-window paradigm. Attention, Perception, & Psychophysics.

[42] Võ, M. L. H. & Henderson, J. M. Does gravity matter? Effects of semantic and syntactic inconsistencies on the allocation of attention during scene perception. *Journal of Vision* **9** (2009). 10.1167/9.3.24 19757963

[43] Gareze, L. & Findlay, J. M. Absence of scene context effects in object detection and eye gaze capture. In *Eye Movements* 617–637 10.1016/B978-008044980-7/50031-8 (2007).

[44] Biederman I (1972). Perceiving Real-World Scenes. Science.

[45] Potter M (1975). Meaning in visual search. Science.

[46] Castelhano MS, Henderson JM (2008). The influence of color on the perception of scene gist. Journal of Experimental Psychology: Human Perception and Performance.

[47] Fei-Fei L, Iyer A, Koch C, Perona P (2007). What do we perceive in a glance of a real-world scene?. Journal of Vision.

[48] Oliva A, Torralba A (2006). Building the gist of a scene: the role of global image features in recognition. Progress in Brain Research.

[49] Castelhano MS, Henderson JM (2003). Flashing scenes and moving windows: An effect of initial scene gist on eye movements. Journal of Vision.

[50] Võ MLH, Henderson JM (2010). The time course of initial scene processing for eye movement guidance in natural scene search. Journal of Vision.

[51] Hollingworth A, Henderson JM (1998). Does consistent scene context facilitate object perception?. Journal of Experimental Psychology General.

[52] Biederman I, Mezzanotte RJ, Rabinowitz JC (1982). Scene perception: Detecting and judging objects undergoing relational violations. Cognitive Psychology.

[53] Loftus GR, Mackworth NH (1978). Cognitive determinants of fixation location during picture viewing. Journal of Experimental Psychology: Human Perception and Performance.

[54] Greene, M. R. Statistics of high-level scene context. *Frontiers in Psychology* 1–31 (2013). 10.3389/fpsyg.2013.00777 PMC3810604 24194723

[55] Land MF, McLeod P (2000). From eye movements to actions: how batsmen hit the ball. Nature neuroscience.

[56] Hayhoe MM, Ballard D (2014). Modeling Task Control of Eye Movements Minireview. Current Biology.

[57] Elazary L, Itti L (2008). Interesting objects are visually salient. Journal of Vision.

[58] Henderson JM, Hayes TR (2018). Meaning Guides Attention in Real-World Scene Images: Evidence from Eye Movements and Meaning Maps. Journal of Vision.

[59] Tatler BW (2007). The central fixation bias in scene viewing: selecting an optimal viewing position independently of motor biases and image feature distributions. Journal of Vision.

